# Supplementary material for: Machine learning approaches for predicting the link of the global trade network of liquefied natural gas
Source: PLoS One. 2025 Jul 30;20(7):e0326952. doi: 10.1371/journal.pone.0326952 (PMC12310025; doi:10.1371/journal.pone.0326952)
Supplement: Attachment — Submitted filename: review_ms_2025_0509.docx [file pone.0326952.s002.docx]

**Detailed Comments to Author**

## Paper Title

## Machine learning approaches for predicting the link of the global trade network of Liquefied Natural Gas

## Background

The authors examine the significant issue of predicting future trade partners in the global Liquefied Natural Gas (LNG) market. As the cleanest burning hydrocarbon, LNG plays a crucial role in the transition to a cleaner energy future. Given the rising geopolitical tensions and the volatility in global LNG trade, it is essential for governments to identify potential trade partners and analyze the structure of global trade networks. Historically, the prediction of future trade relationships has relied on similarity measures such as Common Neighbors (CN) and Adamic-Adar (AA). However, the authors propose an advanced methodology incorporating machine learning algorithms and complex network theory to improve link prediction accuracy within the global LNG trade network from 2001 to 2020.

## Method

The authors combine machine learning techniques with complex network theory to predict links within the LNG trade network. Using local and global similarity-based indices (e.g., CN, Jaccard, Preferential Attachment), the authors apply machine learning algorithms like Random Forest and Decision Trees to predict potential links between nodes. They also introduce Graph Attention Networks (GATs) to incorporate higher-order features such as motif data, which capture more intricate topological patterns. The methodology utilizes these indices to create a feature set that helps the machine learning models predict future links. The model's reliability is validated using the Receiver Operating Characteristic (ROC) curve.

## Materials

The study uses data from the UN COMTRADE database, which includes annual international LNG trade data from 2001 to 2020, focusing on trade volumes between various countries. The authors refine this dataset by excluding countries with zero imports to enhance the quality of the prediction model. This trade data is used to construct the global LNG trade network, modeled as a complex network with countries as nodes and trade relationships as edges. The network is represented as an undirected, weighted graph, where trade relationships are quantified by trade volume, which is crucial for the prediction process.

## Results

The authors demonstrate that local similarity-based indices (such as CN, JA, AA, and PA) when used with machine learning algorithms, particularly Random Forest and Decision Trees, provide strong predictive performance for potential links in the LNG trade network. The results indicate that the Decision Tree and Random Forest algorithms outperform other machine learning models such as Logistic Regression and SVM, based on metrics like accuracy, precision, and the AUC score. The paper highlights that the performance of these algorithms is enhanced by the integration of motif-based features via Graph Attention Networks, which learn from the topological structure of the network. The findings suggest that GATs, by considering both edge and motif information, provide more accurate predictions than traditional models using local similarity indices alone. Additionally, the authors show that global similarity-based indices such as Katz and PageRank did not perform as well as the local similarity measures, indicating that the predictive power of these indices may be limited in the context of LNG trade networks.

## Conclusion

In conclusion, the authors argue that the proposed machine learning-based approach, particularly when incorporating Random Forest and Decision Trees with local similarity indices, is highly effective for predicting potential links within the global LNG trade network. The addition of motif-based data and the use of Graph Attention Networks significantly improve prediction accuracy, surpassing the performance of traditional methods. The authors also acknowledge the limitations of global similarity-based indices and suggest that combining them with local features could improve prediction models in future work. Looking ahead, the authors propose exploring time-series data and dynamic networks to further enhance the prediction model, particularly in the context of evolving geopolitical factors and trade dynamic.

## Comments

1. **Handling Data Sparsity and Imbalance:**

Given that the global LNG trade network involves a diverse set of countries with varying levels of trade volume, how does the model handle potential data sparsity or imbalance in the trade relationships? Are there any specific techniques, such as oversampling, undersampling, or the use of synthetic data, that could be implemented to improve model performance in predicting links for countries with minimal trade interactions?

2. **Generalizability of the Model.**

While the study focuses on the LNG trade network, how do the authors assess the generalizability of the proposed method to other global trade networks, such as oil, minerals, or renewable energy? Are there any adjustments or considerations that need to be made for the model to apply to other commodities with different trade dynamics and structures?

3. **Impact of External Factors on Model Performance.**

In the paper, the authors primarily focus on network topological features for link prediction. However, external factors, such as geopolitical tensions, trade policies, and economic fluctuations, can significantly affect trade relationships. How might the authors incorporate these external factors into the model to improve its robustness and accuracy, particularly during periods of geopolitical instability or economic crises?

4. **Data availability.**

It is recommended to upload and publicly share the entire dataset used in this article, rather than simply providing the name of the database.

## Recommendation

Minor revision
