# Supplementary material for: Machine learning approaches for predicting the link of the global trade network of liquefied natural gas
Source: PLoS One. 2025 Jul 30;20(7):e0326952. doi: 10.1371/journal.pone.0326952 (PMC12310025; doi:10.1371/journal.pone.0326952)
Supplement: Attachment — Submitted filename: Response to Reviewers2.docx [file pone.0326952.s004.docx]

**Response to Reviewer 1:**

1. The conclusion presented in the abstract lacks clarity and fails to effectively summarize the key findings and contributions of the study.

Thanks for your comment, we have added the summary of the key findings in the abstract.
2. Incorporating a literature review table is suggested to clearly highlight the innovations of your research and its contribution to the field.

Thanks for your comment, we have added the literature review table in Related studies.

3. The selection of diverse algorithms such as Random Forest, Decision Tree, SVM, and KNN for comparing their performance in terms of accuracy and predictive capability is very useful. It might be beneficial for the authors to pay more attention to the hyperparameter tuning for each of these algorithms to ensure the use of optimal settings for each model.

Thanks for your comment. Actually, we have tuned the algorithms, but the outperformances were similar. As your suggestions, we have added the demonstraction of the hyperparameter tuning.
4. Although the data for past years has been well presented, it could be interesting to explore how the proposed models would be able to predict new links under future conditions and varying geographical-economic variables.

Thank you for highlighting these valuable factors. Our research team will systematically examine geographical-economic parameters and material price dynamics in our next study to strengthen the model's practical applicability.
5. A section discussing the limitations of the proposed models and the challenges ahead (such as data uncertainties or political-economic changes) could enrich the article further.

Thank you for this important observation. We have enhanced the limitations discussion in Section 4 to better address the study's boundaries and potential extensions
6. The explanations related to the methodology are somewhat brief. It would be beneficial to provide more detailed and in-depth descriptions of the methods used, including specific steps, assumptions, and rationale behind the chosen approaches. This would enhance the clarity and reproducibility of the research.

Thanks so much for your comment, we have listed the methodology steps in the revised manuscript.
7. You may consider adding a "Discussion" section to the paper. This section could provide a deeper analysis of the results, address the limitations of the proposed models, and discuss the implications of the findings in the context of future trade predictions and potential challenges.

We appreciate your comments. Given that the findings and results are closely intertwined, we have combined them into a single 'Results and Discussion' section (section 4). Additionally, we provide an in-depth analysis of the limitations of the algorithms and models used in this study.

**Response to Reviewer 2:**
1. Handling Data Sparsity and Imbalance:

Given that the global LNG trade network involves a diverse set of countries with varying levels of trade volume, how does the model handle potential data sparsity or imbalance in the trade relationships? Are there any specific techniques, such as oversampling, undersampling, or the use of synthetic data, that could be implemented to improve model performance in predicting links for countries with minimal trade interactions?

Reply: Thank you for your review comments. Regarding the potential sparsity and imbalance of trade relationship data, we did not use oversampling, undersampling, or synthetic data because these methods would affect the distribution of the original data. Our method is to standardize and normalize the trade volume. At the same time, since this study mainly focuses on and discusses important nodes in the trade network, our current processing method can meet the requirements.

2. Generalizability of the Model.
   While the study focuses on the LNG trade network, how do the authors assess the generalizability of the proposed method to other global trade networks, such as oil, minerals, or renewable energy? Are there any adjustments or considerations that need to be made for the model to apply to other commodities with different trade dynamics and structures?

Reply: Thank you for your review comments. This study focuses on the liquefied natural gas (LNG) trade network. However, due to our standardized processing of data for deep learning networks, including data normalization and normalization, as well as the construction of node feature matrix, edge matrix, and edge feature matrix suitable for pytorch and torch_geometric, this method is also applicable to other global trade networks (such as oil, mineral or renewable energy trade networks), as other global trade network data can be processed in the same way.

3. Impact of External Factors on Model Performance.
In the paper, the authors primarily focus on network topological features for link prediction. However, external factors, such as geopolitical tensions, trade policies, and economic fluctuations, can significantly affect trade relationships. How might the authors incorporate these external factors into the model to improve its robustness and accuracy, particularly during periods of geopolitical instability or economic crises?

Thank you for your review comments. In future research, we plan to incorporate external factors such as geopolitical tensions, trade policies, and economic fluctuations into the model. We plan to take the following measures: when dealing with unexpected events such as geopolitical tensions and trade policies, we will collect external factors of geopolitical tensions and trade policies from historical events, and use economic analysis methods to identify quantified trade impact values on trade nodes and trade relationships. These values will be input as additional sudden impact factors into the model of this study, so that the model can capture the impact of unexpected events on trade networks.

In response to the impact of economic fluctuations, in the future, this study will collect trade-related economic statistical data and input it into the model of this study after processing, so that the model can capture the impact of economic fluctuations on trade networks.

4. Data availability.
It is recommended to upload and publicly share the entire dataset used in this article, rather than simply providing the name of the database.

Reply, thank you for your review comments. We will submit the data used in this study.
